# Effects of body composition as measured by CT on clinical outcomes in patients with oesophageal cancer

**DOI:** 10.1186/1470-7330-15-S1-P25

**Published:** 2015-10-02

**Authors:** A Mahajan, G Cook, V Goh, C Yip, MM Siddique

**Affiliations:** 1 St Thomas' Hospital, Kings College London, Westminster Bridge Road, London, SE1 7EH, UK

## Aim

The purpose of our study was to evaluate the role of CT-based body composition measurements in predicting clinical outcomes in patients with operable oesophageal cancer.

## Methods

A total of 123 patients who had baseline whole-body 18F-FDG PET/CT scans and underwent NACT followed by surgery for oesophageal cancer were included. Morphometric parameters including skeletal muscle density (SMD), skeletal muscle index, FM, FFM, FMR, VA/SA were measured and were correlated to clinical parameters, surgical findings and pathological chemotherapy response. The factors contributing to DFS and OS were analyzed by univariate and multivariate Cox proportional hazards models.

## Results

76 patients were sarcopenic (62%), including 28 (23%) who had poor muscle quality. Sarcopenic patients were otherwise similar with respect to preoperative BSA, receipt of preoperative chemotherapy, preoperative histopathology, TNM stage, tumour grade, presence of lymphovascular invasion (LVI), circumferential resection margin (CRM) positivity, Mandard score, and response to chemotherapy (P > 0.05 for all). Skeletal muscle mass at the L3 level and total LBM mass were the only parameters which were significantly associated with presence of LVI (p = 0.031). Multivariate analysis indicated that LVI (p = 0.005), Mandard score (p = 0.014), and SMD (p = 0.003) were risk factors for both OS and PFS.

## Conclusion

SMD as an analytic morphometric parameter provided objective data that stratified outcome better than commonly used risk factors such as age, BMI and other comorbidities. We suggest that SMD should be considered as an adjustable variable to be included in the risk stratification of oesophageal cancer patients.

